# Supplementary material for: Emergence and stabilization of a neo-Y chromosome in nematode species with rare males
Source: Mol Biol Evol. 2026 Jul 22;43(8):msag177. doi: 10.1093/molbev/msag177 (PMC13511141; doi:10.1093/molbev/msag177)
Supplement: Supplementary file 1 [file msag177_supplementary_data.pdf]

Supplementary Materials: ‘Emergence and stabilisation of a neo-Y chromosome in nematode species with rare males’

	M. belari	***M. monhystera***	***M. longespiculosa***	***M. spiculigera***
Assembly span (#scaffolds)	241 Mbp (454)	211 Mbp (441)	138 Mbp (725)	147 Mbp (275)
Assembly N50	6.81 Mbp	10.2 Mbp	441 Kbp	10.88 Mbp
%BUSCO genes found (%single-copy, %duplicated)	83.6 (77.8, 5.8)	84.1 (79.9, 4.2)	83.1 (71.8, 11.3)	81.1 (62.2, 16.9)
% autosome-assigned (span, #scaffolds)	63.5 (153.2Mbp, 22)	70.8 (149.4Mbp, 16)	80.9 (111.5 Mbp, 394)	70.7 (103.9 Mbp, 21)
%X-assigned (span, #scaffolds)	6.3 (15.1Mbp, 2)	8.1 (17.1Mbp, 2)	8.32 (11.5Mbp, 20)	15.4 (22.6Mbp, 6)
%Y-assigned (span, #scaffolds)	3.2 (7.8Mbp, 20)	8.1 (17.0Mbp, 152)	NA	NA
%unclear-assigned (span, #scaffolds)	27.0 (65.3Mbp, 410)	13.0 (27.4Mbp, 271)	10.8 (14.9 Mbp, 311)	13.9 (20.5 Mbp, 248)

Table S1. Summary of assembly statistics and how much of each assembly was assigned to X/Y/autosomal/unclear status, for the four focal species of this study.

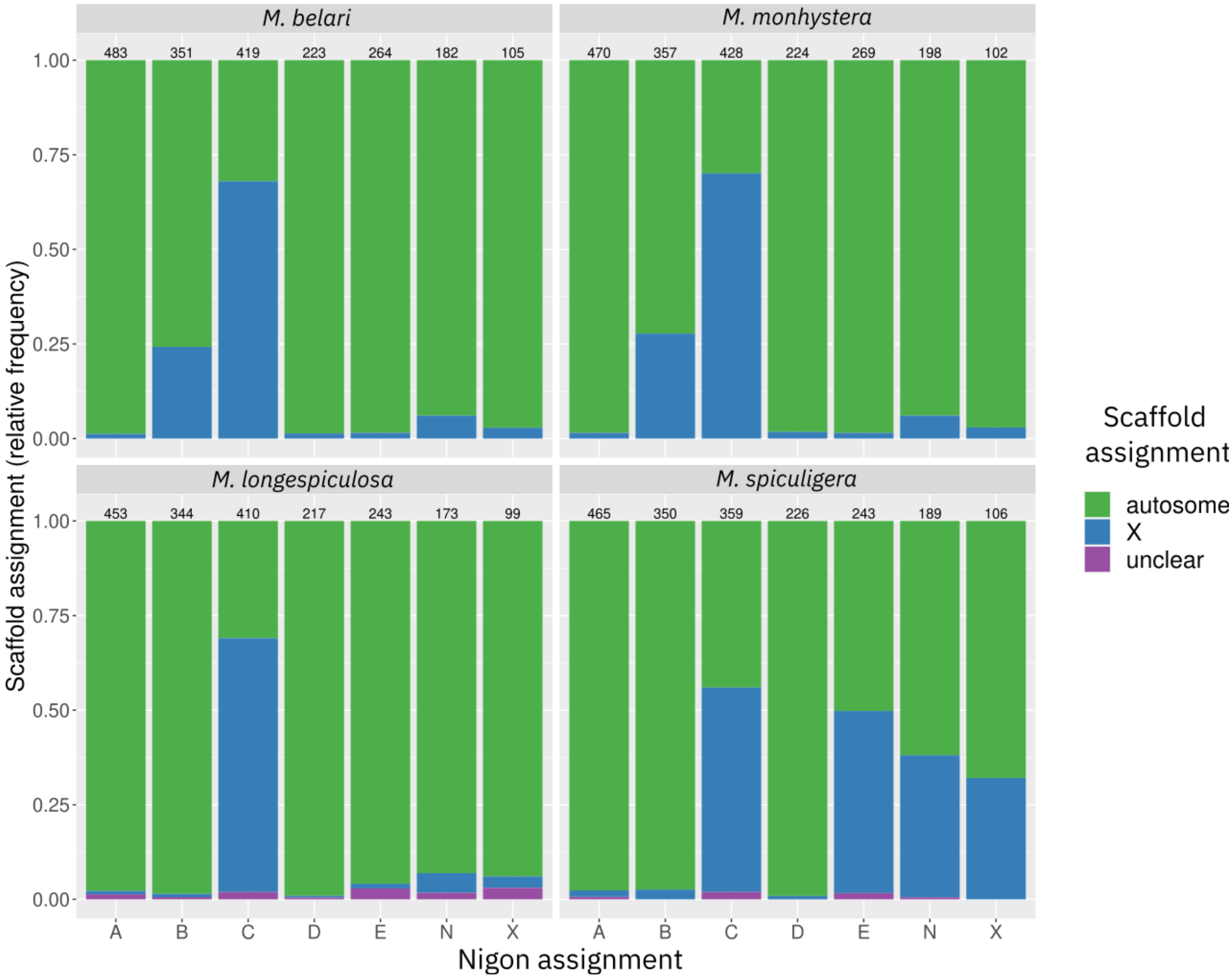

Fig. S1. Average X/autosomal/unclear assignments for each Nigon and each of the four studied species. For each species (panels) and each Nigon (x-axis), the relative distribution of that Nigon's presence on X, autosome - or 'unclear' if the scaffold's assignment was not clear-cut (see Methods) is shown. Numbers above each bar give the total number of each Nigon gene. This allows assessing whether a given Nigon is more or less fully assigned to the X or to autosomes. For all four species, a majority of Nigon C is assigned to the X chromosome. Only part of Nigon B is on the X chromosome in the two pseudosexual species (upper panels); the same holds for Nigons N, X and E for *M. spiculigera* (lower-right panel). Nigon X, the ancestral X in the order Rhabditida (to which *Mesorhabditis* belongs), has become fully autosomal in all but one species here (*M. spiculigera*).

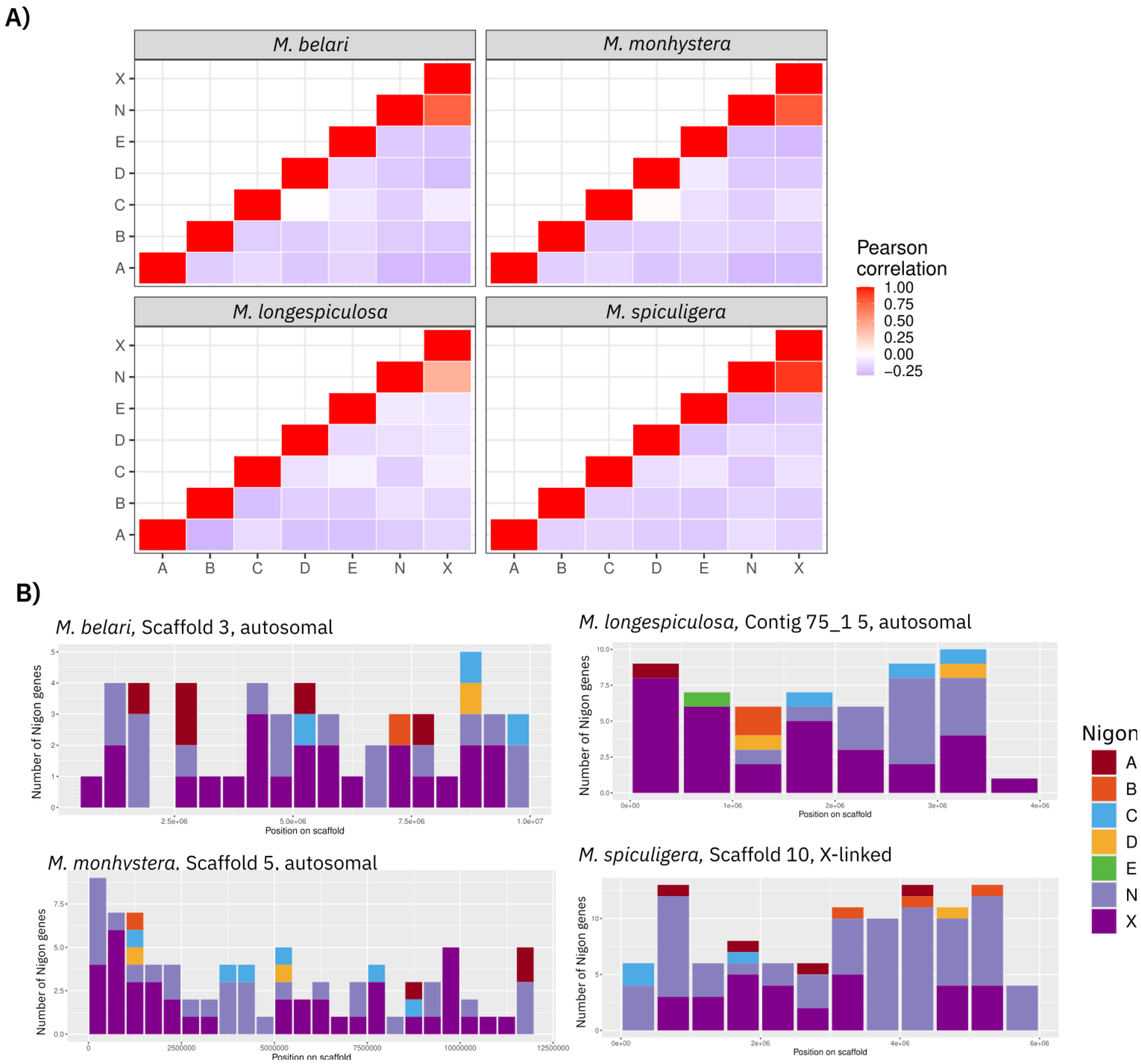

Fig. S2. Analysis of Nigon element linkage and mixing patterns. A) For each scaffold, the relative abundance of each Nigon element was computed. Only scaffolds with at least 10 Nigon genes were included to ensure confident painting. For each Nigon element, a vector storing its relative abundance across all scaffolds was then constructed, and the Pearson correlation coefficient was computed between all pairs of vectors. By construction, a perfect positive correlation is observed on the diagonal (red rectangles). All cross-correlations are near zero, or negative, except for Nigon X and Nigon N genes that are always positively associated on assembled scaffolds. **B)** Example scaffolds containing many Nigon X and N genes. The number of Nigon genes (y-axis) per 500kbp non-overlapping window along a scaffold (x-axis) is shown. Only in *M. spiculigera* are Nigon X/N genes partially X-linked; in the other three species, they are on autosomes (Fig. S1). The Nigon N and X genes appear in mixed order along scaffolds, indicating they have undergone positional shuffling.

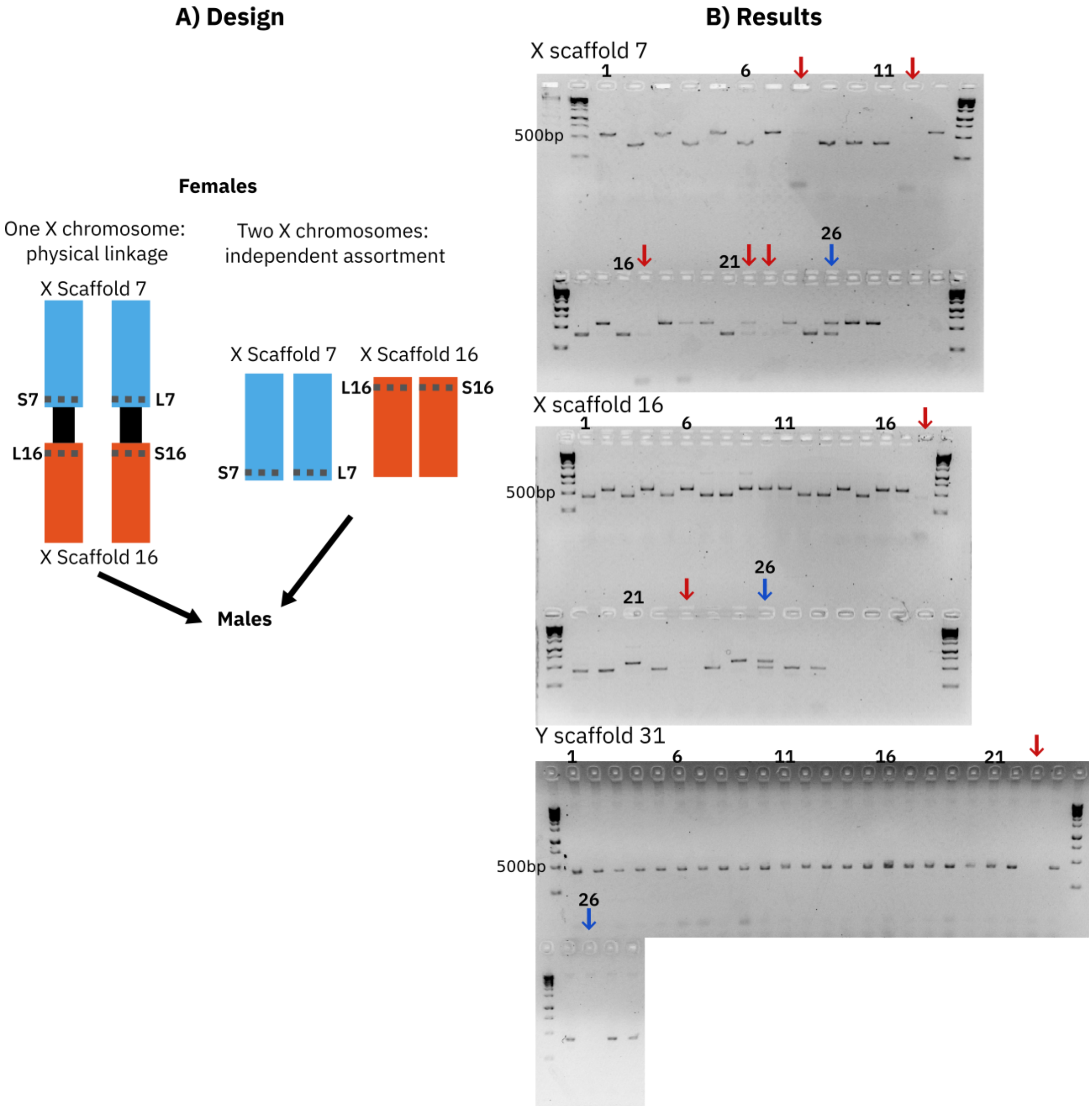

Fig. S3. **PCR-based validation of the X-autosome fusion in *M. belari*. A) Experimental design.** In *M. belari*, two X-assigned scaffolds were identified, scaffold 7 and scaffold 16, coloured here by their majority Nigon assignment (see main text). In females, where two X chromosomes occur, either these two scaffolds are physically linked (left-hand part), or they occur as distinct units (right-hand part, i.e. two X chromosomes in cells). To distinguish these, we identified heterozygous indels on each scaffold, near their extremities, shown as dotted lines. 'S7' means 'short allele on scaffold 7', 'L7' means 'long allele on scaffold 7', and similarly for scaffold 16. We then genotyped males descended from a single female, in which a single copy of each X chromosome is expected. If the two scaffolds are distinct units, the four possible combinations of the two indels should occur at similar frequencies (25%), due to independent assortment under Mendelian segregation. If the two scaffolds are physically linked, two of the four combinations should be disproportionately observed, the other two being produced by recombination events. **B) Results.** 27 males and one female were lysed and PCR amplified for each X chromosome indel, plus a Y chromosome locus (on scaffold 31, see Methods). Numbers above wells refer to genotyped individuals. The female is shown with a blue arrow (well number 26), and amplifies two bands at the X loci - confirming the locus is heterozygous - but does not amplify the Y locus, as expected. For the remaining wells, males amplified a single band at the Y marker, confirming they were male, and a single band at the same size as one of the two female bands for the two X markers. The expected sizes are: S7, 395bp; L7, 577bp; S16, 437bp; L16, 541bp;

Y scaffold 31, 436bp. To the left, the ladder band at size 500bp is shown. For wells marked with red arrows, bands were too faint and those individuals were discarded from the analysis. For the remaining 21 males, the following combinations of X genotypes were observed: 10 S_7L_{16}, 11 L_7S_{16}, 0 S_7S_{16}, 0 L_7L_{16}. Thus physical linkage is clearly supported; see Methods for statistical support.

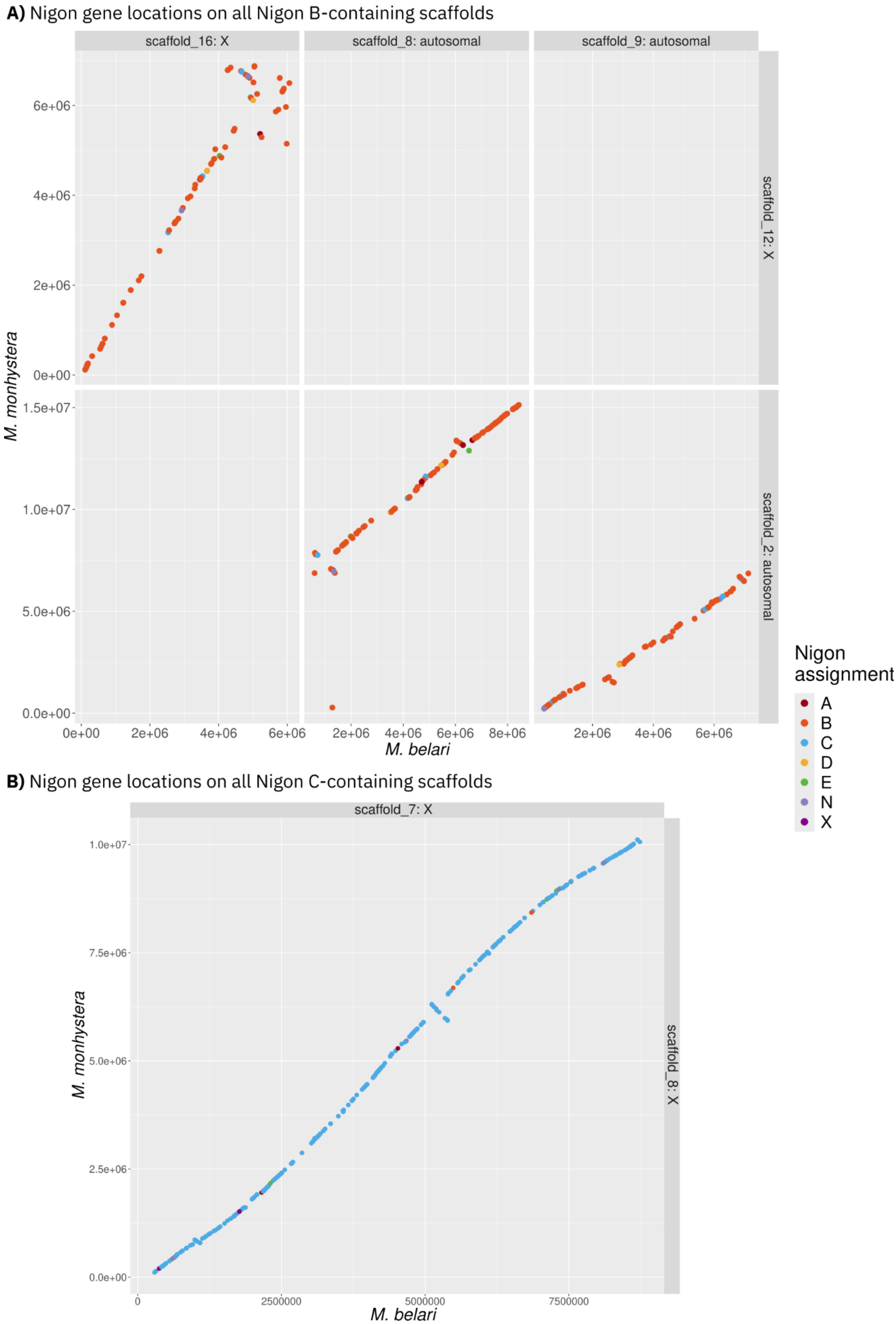

Fig. S4. Nigon genes are conserved and co-linear on the X-linked scaffolds of the two pseudosexual species. In both pseudosexual species, *M. belari* and *M. monhystera*, the X

chromosome is composed of two scaffolds, each mostly composed of Nigon B (orange) and Nigon C (blue) (Fig. 4B). To test for a common evolutionary origin of these X-linked scaffolds between the two species, we extracted all scaffolds mostly composed of Nigon B and Nigon C genes, and compared the positions on these scaffolds (x and y axes) of the Nigon genes found across both species. **A)** Results for Nigon B. The X-linked scaffolds share the same Nigon B genes along their entire lengths, in conserved positional order, except for one inversion. This also holds for the autosomal scaffolds, with the autosomal scaffold 2 in *M. monhystera* covering the two autosomal scaffolds 8 and 9 in *M. belari*, along their full lengths. As Nigon B genes are only found on autosomes in the other *Mesorhabditis* species we studied (Fig. S1), this strongly suggests that part of an ancestral Nigon B autosome fused with the X chromosome, in the ancestor of *M. belari* and *M. monhystera*. **B)** Results for Nigon C. The Nigon C genes are also shared across the entire scaffold lengths and in conserved positional order, except for one small inversion.

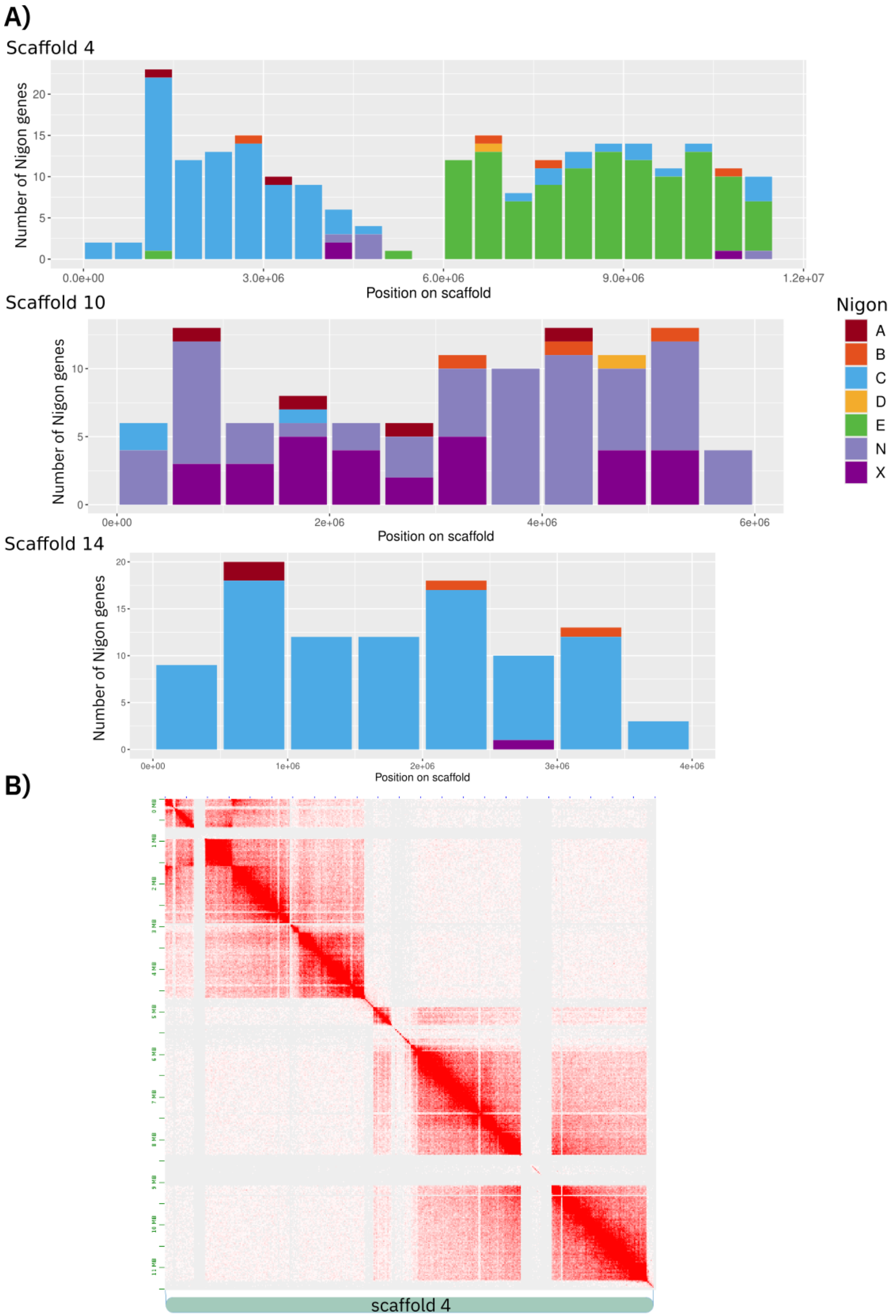

Fig. S5. Nigon composition of the X-linked scaffolds in *M. spiculigera*. A) The three main X-assigned scaffolds in *M. spiculigera* are shown. Each is painted by the number of Nigon genes (y-axis) per 500kbp non-overlapping window along the scaffold (x-axis). From top to bottom, the first

X-assigned scaffold is composed of Nigon C (blue) and E (green), the second of Nigon N (light purple) and X (dark purple), and the third of Nigon C (blue). These three scaffolds are separated by unbridged repeat arrays (Fig. 4B and paper Methods). Extensive reshuffling, visible as bars composed of mixtures of elements, has occurred between Nigon N and Nigon X genes, but not between the other elements. **B)** Hi-C map for scaffold 4 (the uppermost scaffold in panel A), with red dots showing contacts along the scaffold's length. Portions with lower density of red dots, on the diagonal, correspond to repeated sequences. Despite this, the map is consistent with a single, well-assembled physical unit, supporting a true fusion event.

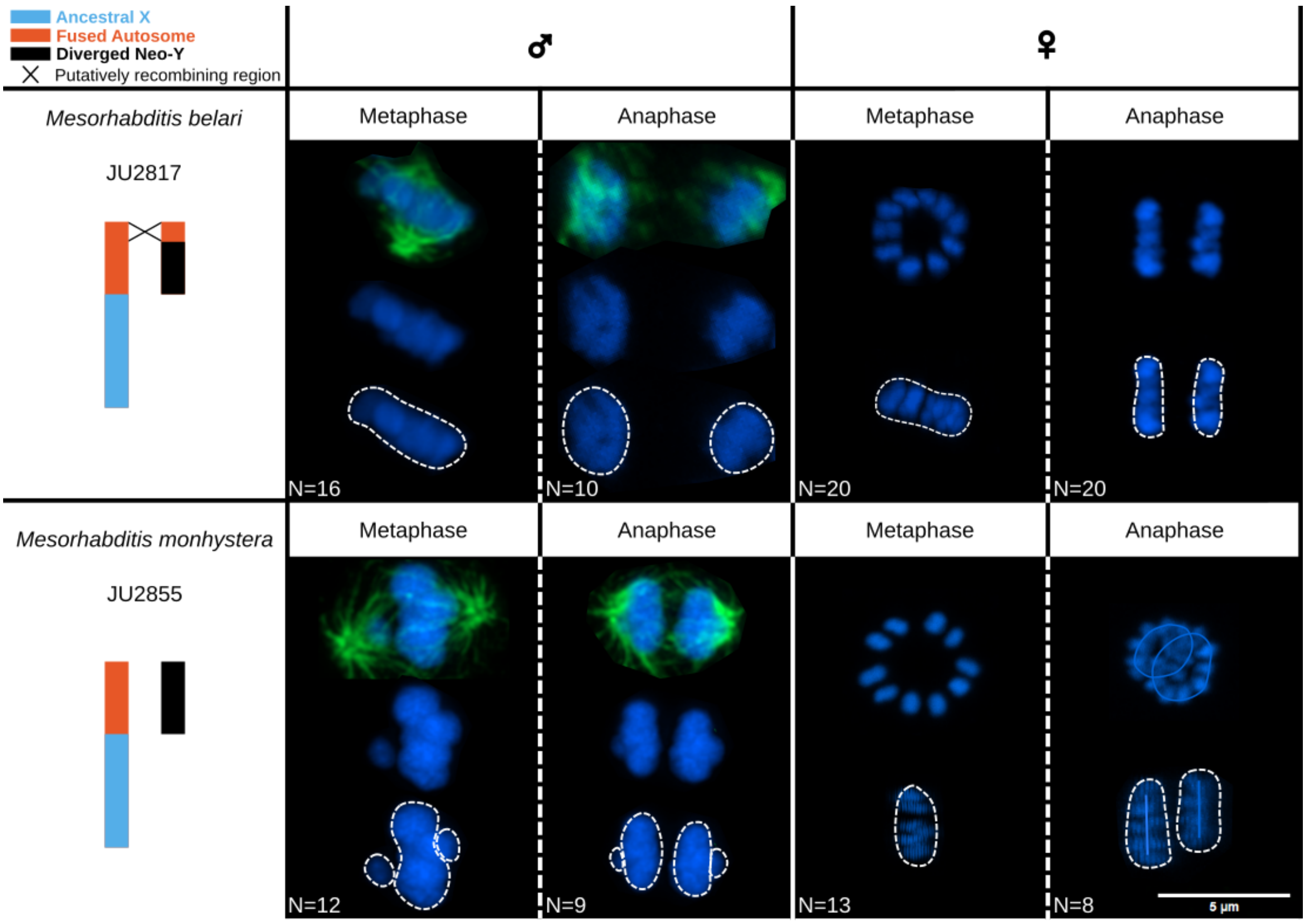

Fig. S6. Two lagging chromosomes observed during male meiosis I in *M. monhystera*. On the left, the X and neo-Y chromosomes are depicted, as in Fig. 5B. The diploid region in *M. belari* is shown, that putatively still recombines. On the right, representative cells in metaphase or anaphase of meiosis I are shown, from *M. belari* (upper panels) and *M. monhystera* (lower panels), in male (left-hand panels) and female (right-hand panels) gonads. DNA is in blue (Hoechst) and tubulin in green (immunostaining using an antibody against alpha-tubulin). White dotted lines show manual delimitation of the DNA content. In male gonads of *M. belari* and female gonads of both *M. belari* and *M. monhystera*, a single mass of DNA is found in metaphase I, and two masses of DNA in anaphase I, as expected if all chromosomes are properly paired. In both species, a characteristic 'rosette' of 10 sets of paired chromosomes can be seen in female metaphase I. By contrast, in male gonads of *M. monhystera*, two additional smaller masses of DNA can be found, in both metaphase I and anaphase I. Because these were never found in males of *M. belari*, nor in females of *M. monhystera* - whose only difference is possessing two X chromosomes that should be paired - we propose these are the X and Y chromosomes of *M. monhystera*, and that the X and Y are unpaired during male meiosis I in this species. In each panel, the number of observed cells (N=) that showed this pattern is provided.

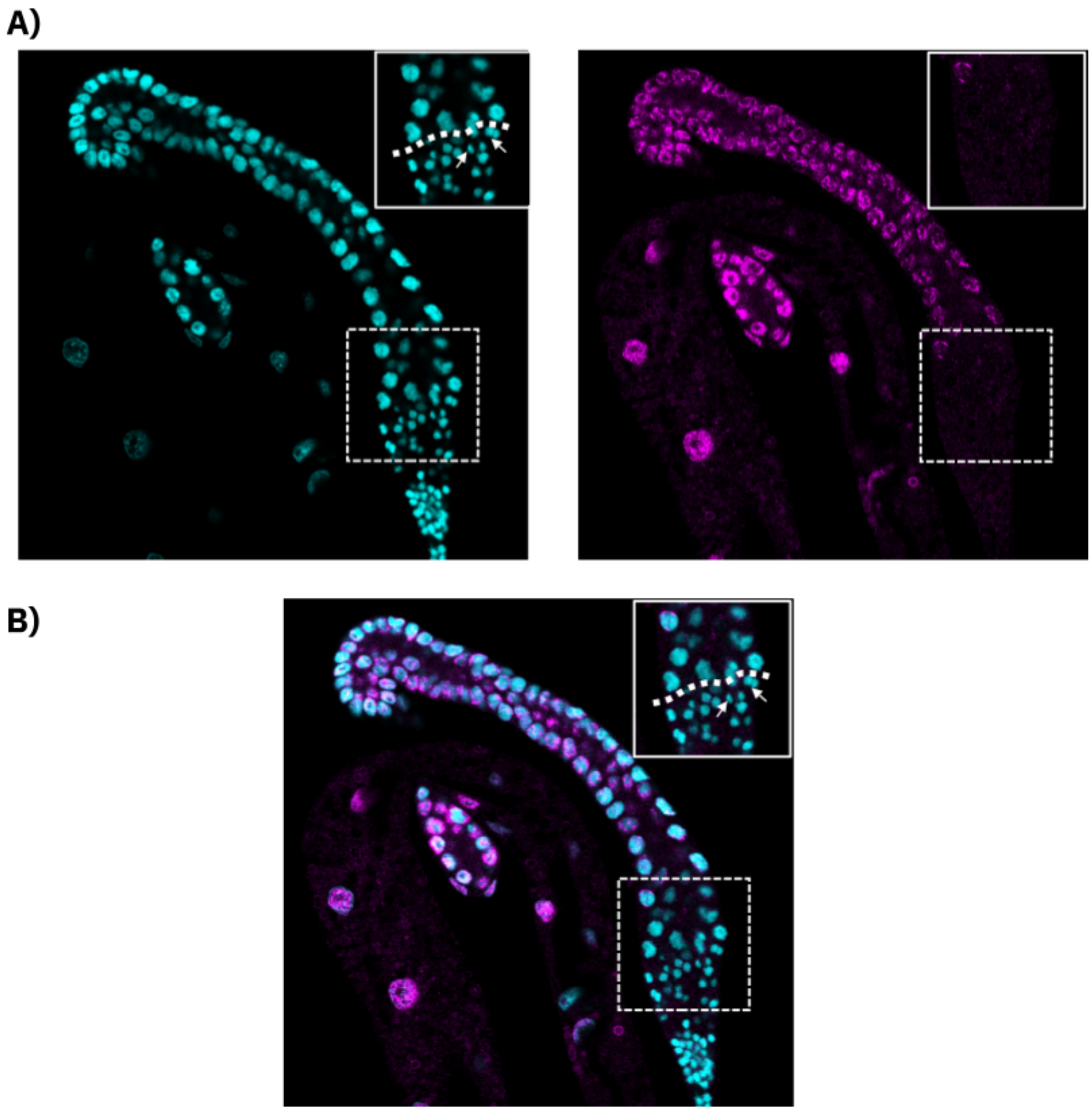

Fig. S7: Spermatocytes are transcriptionally silent from the first meiotic division. A) Gonad of a *M. belari* male, with DNA in cyan blue (left-hand panel) and active transcription in magenta (right-hand panel), as measured using immunostaining with an antibody against the phosphorylated form of Serine 10 on RNA Polymerase II. The gonad is spatially organised: the distal part, top left, contains the spermatocytes in meiotic prophase, which progressively enter the division phases. The proximal part (bottom right) contains the mature spermatids recognisable by their compact DNA. The dividing sperm cells are shown with the white arrows (metaphase and anaphase figures) and the transition is delineated with a white dotted line in the insets. The RNA Pol II signal is no longer detectable from the onset of the meiotic divisions. **B)** Merge of the DNA and RNA Pol II channels.

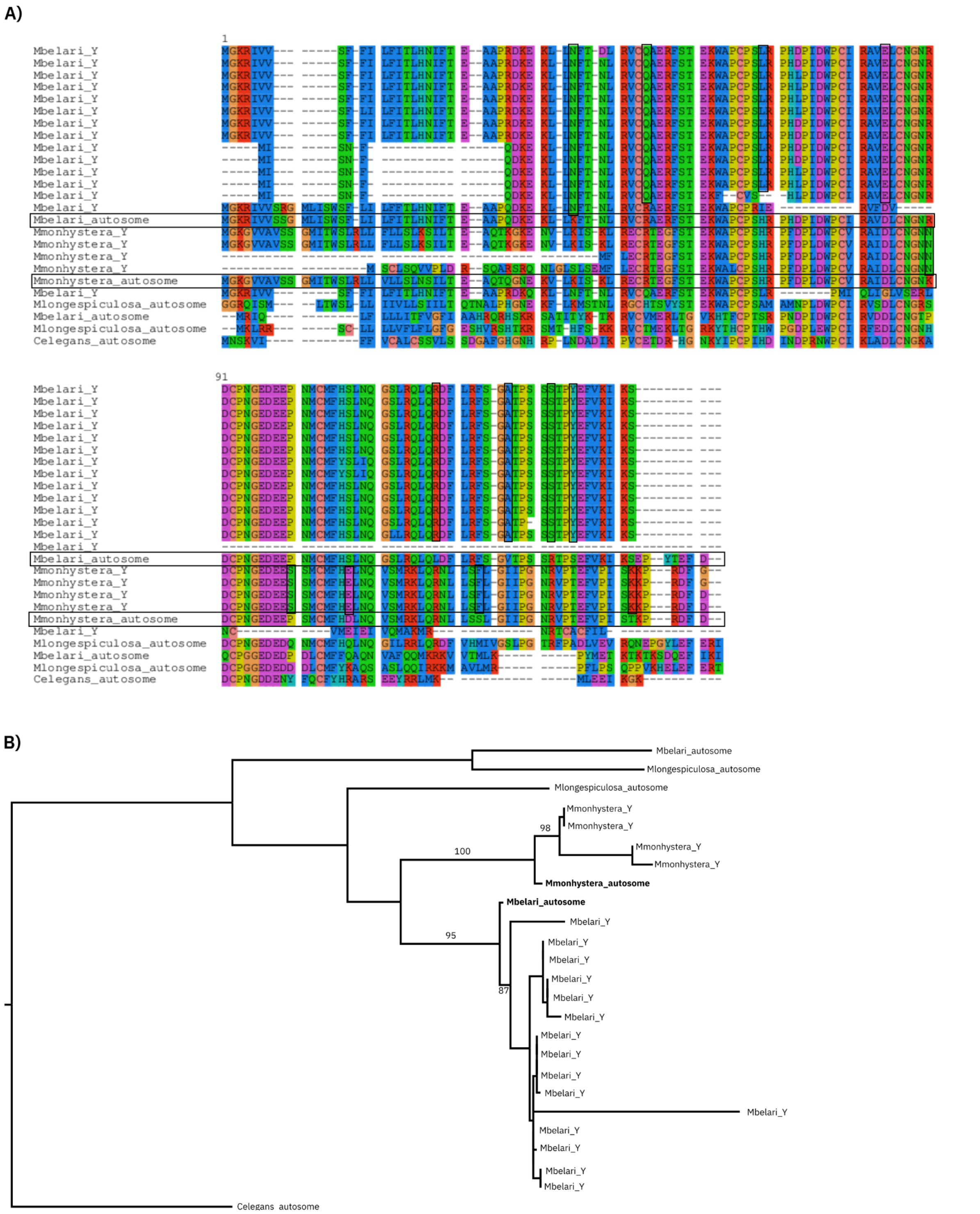

Fig. S8: Evolution of the hen-1-like family of orthologs. A) Multiple sequence alignment of the members of the hen-1-like family. On the left, the species in which the gene was found, and the chromosome it was found on, are indicated. In *M. belari*, 14 Y copies and two autosomal copies are found, and in *M. monhystera*, 4 Y copies and one autosomal copy was found. The *C. elegans* protein *hen-1* is shown in the last row of the alignment. The horizontal boxes highlight the autosomal copies from which the Y copies derived, and the vertical boxes highlight amino-acid substitutions that occurred specifically on the Y copies compared to the autosomal ancestor. These substitutions are specific to each of the two species. The full multiple-sequence alignment is available from Zenodo (see data availability section). **B)** Phylogenetic tree built from the multiple sequence alignment, rooted on the *C. elegans* hen-1 gene. Numbers on branches indicate bootstrap support, as a percentage out

of 1000 bootstrapped trees. Two distinct clades with hen-1-like genes on the Y can be seen, one for *M. belari* and one for *M. monhystera*, each with strong bootstrap support. Within each clade, the autosomal ancestor of the Y-specific duplicated copies is marked in bold; the autosome-Y splits also have strong bootstrap support.

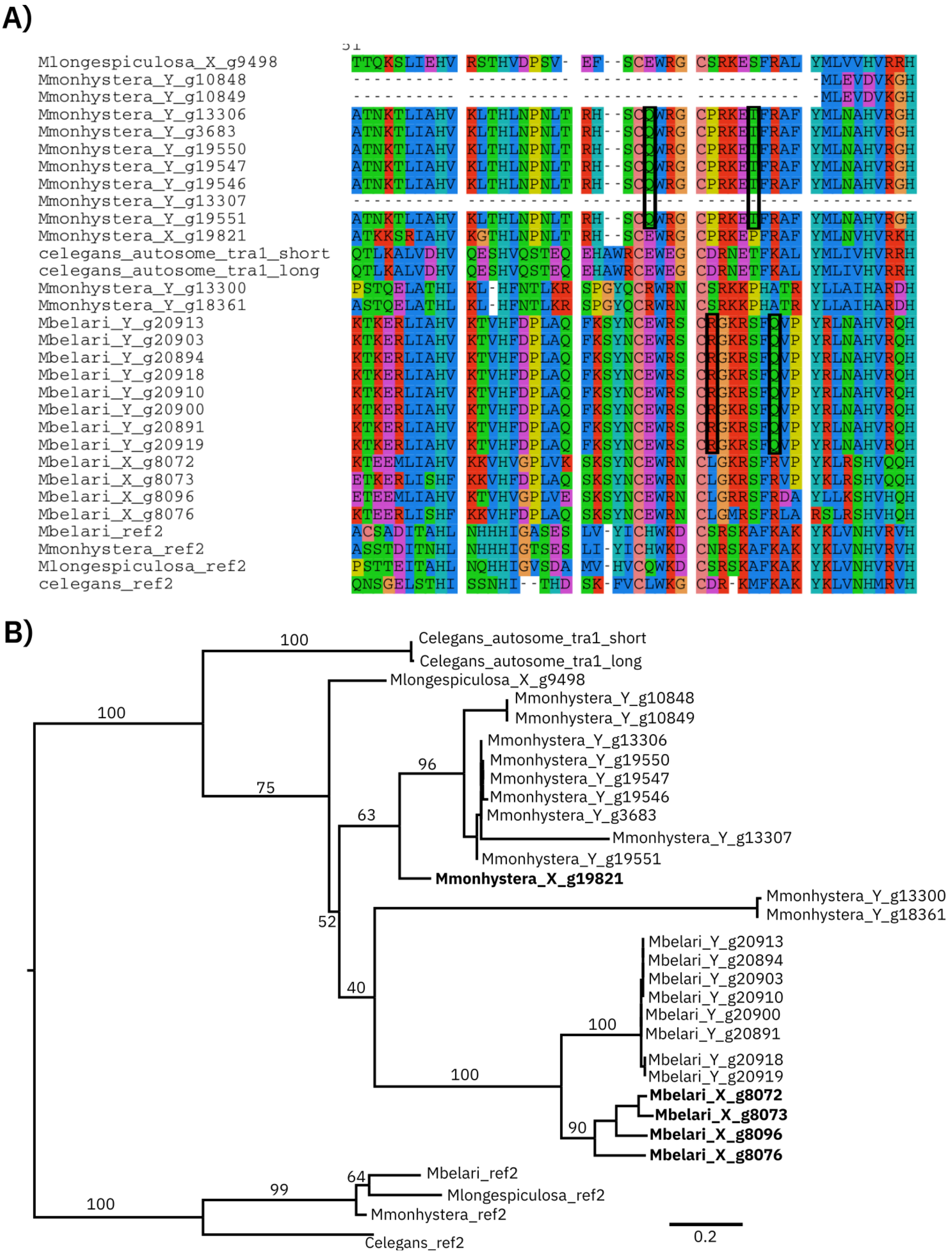

Fig. S9: Evolution of the *tra-1*-like family of orthologs. A) One region of the multiple sequence alignment of the members of the *tra-1*-like family is shown, spanning one of the conserved zinc-finger domains of *C. elegans tra-1* (sequence: 'WRCEWEGCDRNETFKALYMLIVHVRRH', annotated as a zinc finger in Uniprot entry P34708). The *C. elegans* long and short isoforms of *tra-1* are both

included. The *C. elegans* copy is autosomal, while the *M. monhystera* and *M. belari* copies are on the X and neo-Y chromosomes. The vertical rectangles mark positions where the copies on the Y have acquired specific substitutions compared to the copies on the X chromosome. These substitutions are species-specific. Another, tra-1-like zinc finger gene in *C. elegans*, *ref-2*, and its single-copy identified orthologs in *M. belari*, *M. monhystera* and *M. longespiculosa* were included in the alignment, and used to anchor the phylogenetic tree. The full multiple-sequence alignment is available from Zenodo (see data availability section). **B)** Phylogenetic tree built from the full multiple sequence alignment, with the copies on the X marked in bold. The tree was rooted using the branch leading to the *ref-2* genes, as these are expected to be phylogenetically distinct from the *tra-1*-like genes. Numbers on branches indicate bootstrap support, as a percentage out of 1000 bootstrapped trees. The *tra-1*-like clade is entirely distinct from the *ref-2*-like clade, with maximal bootstrap support (100), confirming the *tra-1*-like genes we identified are true orthologs. For both *M. belari* and *M. monhystera*, the X and Y copies are also clearly separated with strong bootstrap support (96 and 100), The scale bar (bottom right) is in amino acid substitutions per site.

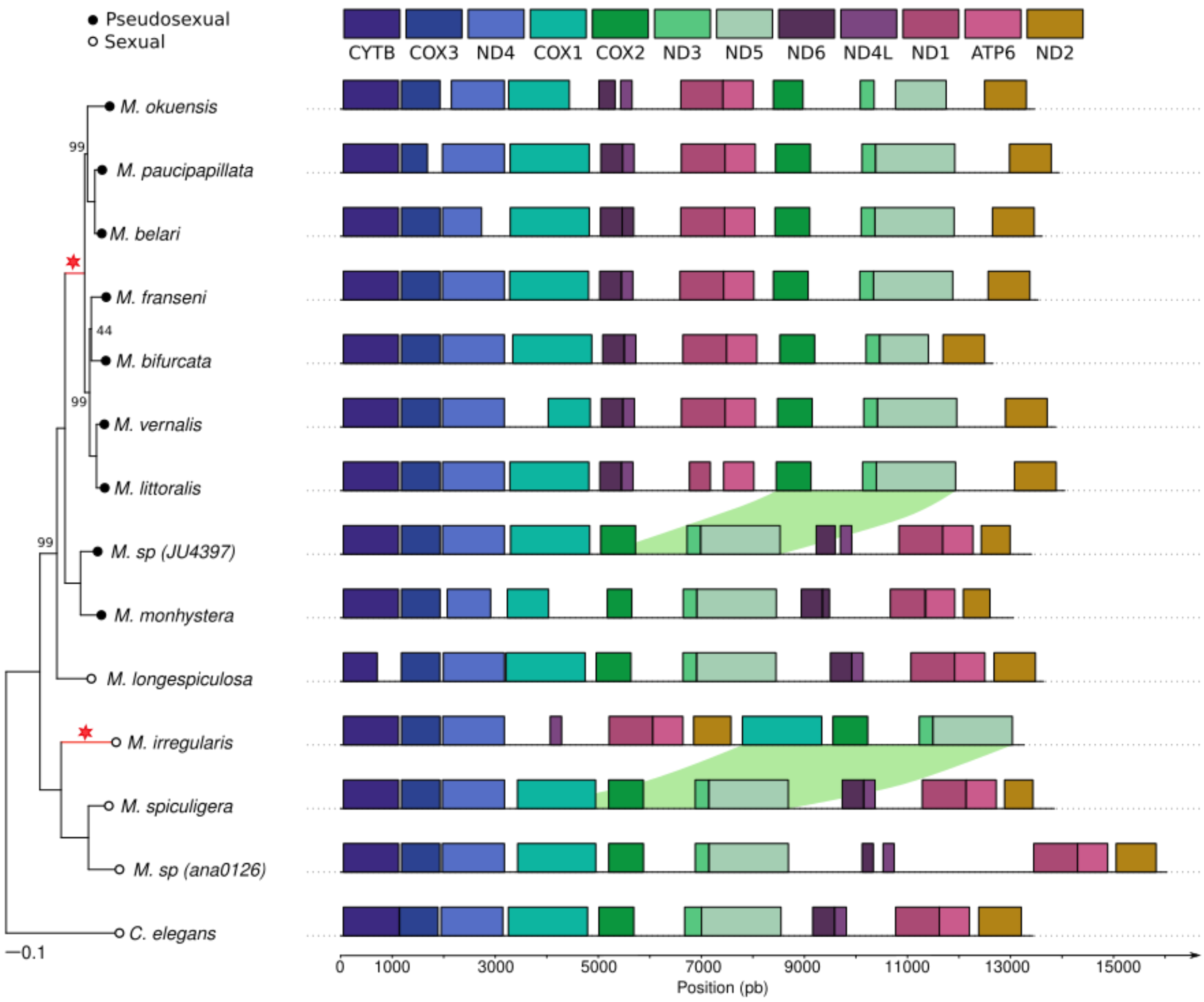

Fig. S10. Two mitochondrial rearrangements have occurred in *Mesorhabditis*. On the left, a phylogeny built from the mitochondrial genomes of our *Mesorhabditis* species, plus *C. elegans* as an outgroup, is shown. The two red stars label branches along which a mitochondrial rearrangement has occurred. Nodes are labelled based on mode of reproduction (filled and open circles). On the right, each mitochondrial gene is shown along the assembled mitochondrial genome of each species (coloured rectangles). The two rearrangements - which are translocations - are highlighted using green shadowing.

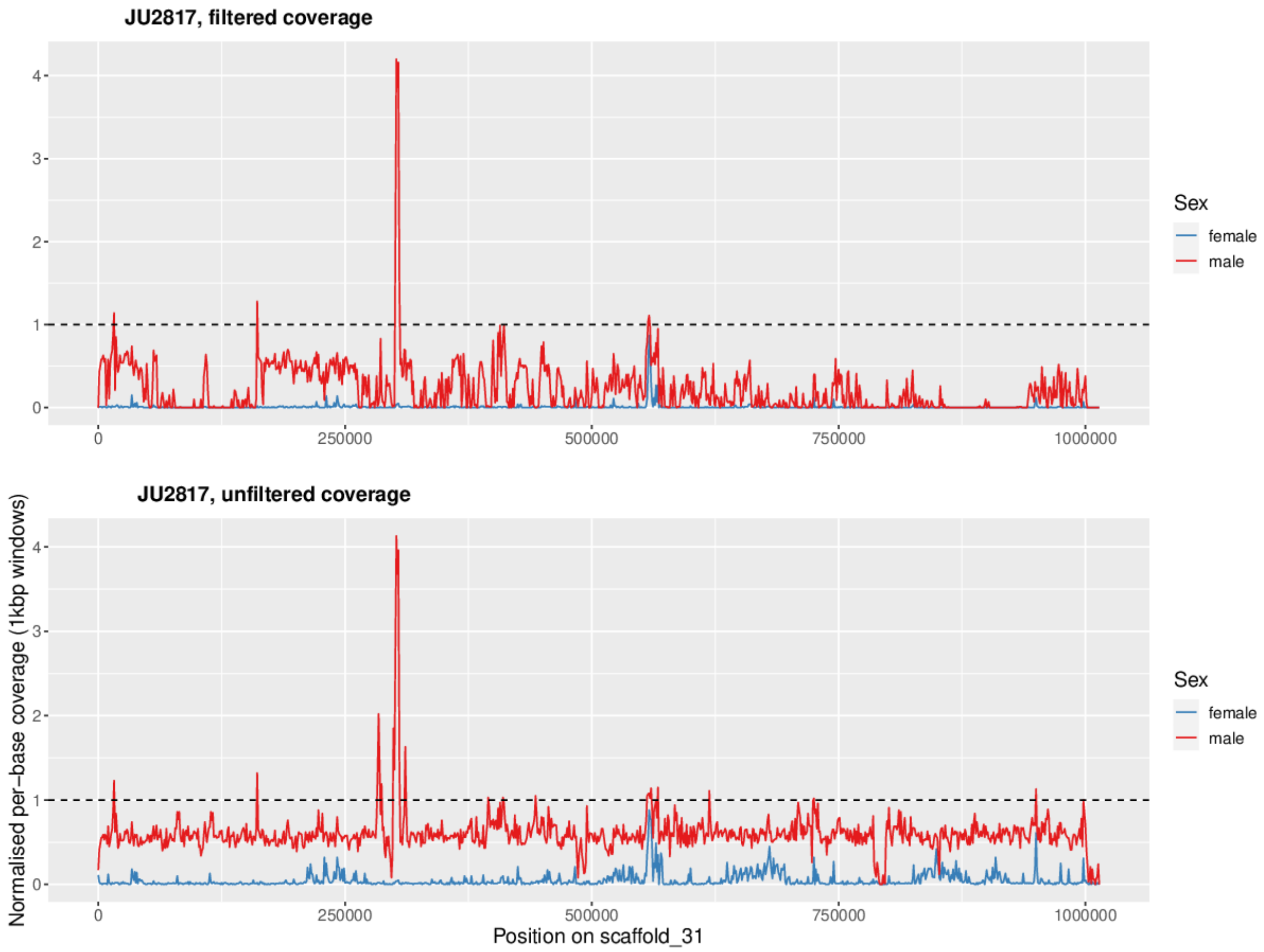

Fig. S11. **Filtered and unfiltered coverage on a Y-assigned scaffold in *M. belari*.** Normalised coverage for male and female DNA-Seq is shown (y-axis) along 1kbp non-overlapping windows of scaffold 31 (x-axis) of the *M. belari* assembled genome. The upper panel shows filtered coverage (MAPQ>=20 + reads are not marked as supplementary, secondary or duplicated), while the lower panel shows coverage but without the MAPQ filter (called 'unfiltered' here). Unfiltered coverage shows the expected haploid (values ~0.5) for males, but also shows some coverage in females. By contrast, filtered coverage shows almost no coverage in females, enabling more confidently assigning this scaffold to the Y. The peak in males probably corresponds to a duplicated region, while the region with filtered coverage in females (upper panel) may correspond to an assembly error.

Scaffold 1	Scaffold 2	Contact type	Contact count	Ratio of inter- to intra-chromosomal contact	Contact counting approach
**scaffold_7**	**scaffold_16**	3prime-3prime	130	0.3	pairix
scaffold_8	scaffold_14	3prime-5prime	100	0.265	pairix
scaffold_14	scaffold_23	5prime-5prime	30	0.073	pairix
scaffold_14	**scaffold_16**	5prime-5prime	24	0.065	pairix
scaffold_8	**scaffold_16**	3prime-5prime	13	0.047	pairix
scaffold_5	**scaffold_16**	5prime-5prime	12	0.045	pairix

Table S2: Top inter-scaffold Hi-C contact counts for *M. belari*. Each row shows contacts computed between the extremities of two different scaffolds (first two columns), the kind of extremities

the contacts are between (5prime or 3prime for start or end of the scaffolds - third column), the number of contacts (fourth column), the ratio of this number to the mean number of contacts inside each of the two extremities (fifth column), and the contact counting approach (see Methods for meaning). The top 10 inter-scaffold contacts, with a minimum of 10 contact counts, were selected and shown here. The X-assigned scaffolds are highlighted in bold. The highest inter-scaffold contacts are between the two X-assigned scaffolds of *M. belari*, however, when removing contacts occurring in duplicated regions of scaffolds, no contacts remain, as no rows with 'contact counting approach' set to 'pairtools' remain.

Scaffold 1	Scaffold 2	Contact type	Contact count	Ratio of inter- to intra-chromosomal contact	Contact counting approach
scaffold_7	scaffold_9	5prime-5prime	54	0.137	pairix
scaffold_9	scaffold_10	3prime-3prime	40	0.084	pairix
scaffold_7	scaffold_13	3prime-5prime	34	0.081	pairix
scaffold_9	scaffold_13	5prime-3prime	39	0.059	pairix
**scaffold_8**	**scaffold_12**	5prime-3prime	127	0.028	pairix
scaffold_3	scaffold_13	3prime-3prime	13	0.019	pairix
scaffold_1	scaffold_15	3prime-3prime	36	0.001	pairix
scaffold_1	scaffold_4	3prime-5prime	29	0	pairix
scaffold_1	scaffold_6	3prime-5prime	23	0	pairix
scaffold_1	scaffold_3	3prime-5prime	12	0	pairix
scaffold_9	scaffold_10	3prime-3prime	14	0.121	pairtools_mapq1
scaffold_9	scaffold_13	5prime-3prime	12	0.063	pairtools_mapq1
**scaffold_8**	**scaffold_12**	5prime-3prime	48	0.034	pairtools_mapq1
**scaffold_8**	**scaffold_12**	5prime-3prime	45	0.032	pairtools_mapq20

Table S3: Top inter-scaffold Hi-C contact counts for *M. monhystera*. For the meaning of the columns, see table above. The X-assigned scaffolds are highlighted in bold. Contacts between the two X-assigned scaffolds are consistently high. In particular, when filtering out contacts occurring in duplicated regions of scaffolds (i.e. looking only at rows with 'contact counting approach' set to 'pairtools'), and with this filtering threshold set to a high value (MAPQ of 20, meaning the probability that a contact is a false positive is <~1%), only contacts between the two X-assigned scaffolds remain. These are all in one orientation - '5prime-3prime' -, consistent with the two scaffolds being part of a single contiguous X chromosome.

	X	Y	autosome
#orthogroups	1521	56	10927
# uniquely assigned orthogroups (% of all orthogroups)	1415 (93%)	12 (21%)	10223 (94%)
#genes	1549	139	13857
#genes/orthogroup	1.02	2.48	1.27

gene density per kbp	0.102	0.018	0.090

Table S4: Gene- and gene family-level statistics for *M. belari*. Columns show X-, Y- and autosome-assigned genes. An orthogroup is a family of genes as defined using OrthoFinder (see Methods), and can include genes assigned to both X, Y and autosomes. #orthogroups is a count of orthogroups with at least one X, Y or autosome-assigned gene member. # uniquely assigned orthogroups counts orthogroups with only members assigned to X, Y or autosomes.

	X	Y	autosome
#orthogroups	1677	79	10189
# uniquely assigned orthogroups (% of all orthogroups)	1567 (93%)	19 (24%)	9771 (96%)
#genes	1721	246	13488
#genes/orthogroup	1.03	3.11	1.32
gene density per kbp	0.101	0.014	0.090

Table S5: Gene- and gene family-level statistics for *M. monhystera*. For table legend, see table above.

	M. belari	*M. monhystera*
X: span, %covered by repeats	15.1 Mbp, 22.2%	17.1Mbp, 28.0%
Y: span, %covered by repeats	7.8Mbp, 70.2%	17.0Mbp, 88.9%
autosomes: span, %covered by repeats	153.2Mbp, 34.0%	149.4Mbp, 32.7%

Table S6: The Y chromosome is enriched for repeats compared to the X and autosomes in the pseudosexual species. For the two pseudosexual species, the span (total length) of all X, Y and autosomal-assigned scaffolds is shown (rows), together with the fraction of those scaffolds covered by repeated elements (Transposable elements, Satellites and other uncharacterised repeated elements) as predicted using EarlGrey (see Methods).
